# Strategies for discontinuation of proton pump inhibitors (PPIs) in patients with long-term PPI administration: a randomized controlled trial

**DOI:** 10.1186/s12876-021-02086-9

**Published:** 2022-01-15

**Authors:** Mariko Hojo, Daisuke Asaoka, Yuji Shimada, Shuko Nojiri, Akihito Nagahara

**Affiliations:** 1 Department of Gastroenterology, Juntendo University School of Medicine, 2-1-1 Hongo Bunkyo-ku, Tokyo, 113-8421 Japan; 2 Department of Gastroenterology, Juntendo Tokyo Koto Geriatric Medical Center, 3-3-20 Shinsuna Koto-ku, Tokyo, 136-0075 Japan; 3 Department of Gastroenterology, Juntendo University Shizuoka Hospital, 1129 Nagaoka, Izunokuni, Shizuoka 410-2295 Japan; 4 Medical Technology Innovation Center, Juntendo University, 2-1-1 Hongo Bunkyo-ku, Tokyo, 113-8421 Japan

**Keywords:** Proton pump inhibitors, Long-term PPI administration, Discontinuation

## Abstract

**Background:**

Proton pump inhibitors (PPIs), including potassium ion-competitive acid blocker, are widely used worldwide and are often used for long periods of time. However, in recent years, potential side effects associated with long-term PPI use have been reported. Many patients take PPI for a long period of time, even though it is unnecessary, and it is necessary to discontinue PPI administration in such patients. However, sudden discontinuation may cause symptoms to recur and discontinuation may be unsuccessful. A strategy for safe and secure PPI discontinuation has not yet been established. The purpose of this study is to determine whether PPI can be safely discontinued by tapering the PPI dose or by abrupt discontinuation of PPI, and to establish a strategy for safe and secure PPI discontinuation.

**Methods:**

The evaluation will be conducted as a multicenter, randomized, parallel-group clinical trial with five assessment points at the start of the study and 2 weeks, 4 weeks, 6 months, and 12 months after the start of the study. One intervention group is the group in which PPI administration is abruptly discontinued (Group A), and the second group is the group in which the PPI dose is gradually tapered and then PPI administration is discontinued (Group B). The primary outcome and secondary outcome are the proportion of patients who successfully discontinued the PPI at 6 months and at 12 months after the start of the study in groups A and B, respectively.

**Discussion:**

We predict that the proportion of patients who successfully discontinue PPI will be higher in the group in which PPI administration was gradually tapered than in the group in which PPI administration was abruptly discontinued. On the other hand, we expect that many participants will succeed in discontinuing PPI regardless of the discontinuation strategy due to the explanation that discontinuation is necessary.

***Trial registration*:**

Japan Registry of Clinical Trials, jRCT1031180383. Registered 20 March 2019, https://jrct.niph.go.jp/latest-detail/jRCT1031180383.

## Background

Proton pump inhibitors (PPIs), including potassium ion-competitive acid blocker (PCAB), have been used to treat gastric ulcer, duodenal ulcer, and reflux esophagitis [1–3]. PPIs are also used to suppress the recurrence of gastric or duodenal ulcer when low-dose aspirin or a nonsteroidal anti-inflammatory drug is administered [4, 5], and to eradicate *Helicobacter pylori* (*H. pylori*) [6]*.* Furthermore, a PPI is used to treat not only mucosal damage such as reflux esophagitis and ulcer, but also non-erosive gastroesophageal reflux disease (NERD) and functional dyspepsia (FD) [7, 8]. The goal of treatment in patients with NERD or FD is symptom control. Therefore, it is possible to discontinue PPI treatment if their symptoms improve or disappear by PPI treatment. In addition, mild esophagitis with Los Angeles grade A or B does not progress to severe esophagitis in 90% of the cases [9]; therefore, it is known that treatment does not need to be continued if there are no symptoms. However, in clinical practice, when prescribed for patients with mild reflux esophagitis or NERD, PPI is often given continuously even if their symptoms disappear.

In recent years, potential side effects associated with PPI use have been reported. Long-term PPI use may affect nutrient absorption, including calcium malabsorption, and increase the risk of fracture [10, 11]. PPI use may increase the risk of enteric infections such as infection with *Clostridium difficile* and *Campylobacter*, and community-acquired pneumonia [12, 13]. The gut microbiota plays an important role in host resistance against colonization by exogenous enteric microbes and overgrowth of indigenous commensals [14]. Two large cohort studies found that PPIs altered the composition of the gut microbiota [15, 16]. Accordingly, the increased risk of enteric infections in PPI users may be caused by the influence of PPI on the gut microbiota. Moreover, intestinal bacterial overgrowth promotes bacterial translocation [17]; therefore, PPIs may cause bacterial translocation.

Therefore, it is necessary to discontinue PPI use in patients who have been taking PPI for a long period of time if PPI is not necessary. However, sudden discontinuation may cause symptoms to recur and discontinuation may be unsuccessful. A strategy for secure PPI discontinuation is being investigated, but it has not yet been established [18]. In this study, we will compare patients in whom the PPI is abruptly discontinued and patients in whom the PPI is gradually tapered, to study which method of PPI discontinuation results in safe and secure PPI discontinuation and to establish a strategy for safe and secure PPI discontinuation.

## Methods/design

### Study design

**Fig. 1:**
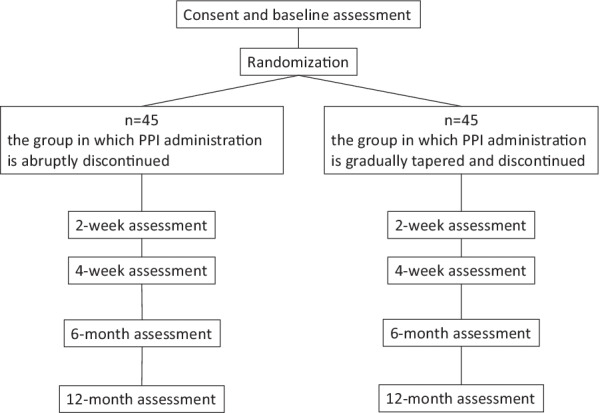
A schematic of the trial design. PPI, proton pump inhibitor

The current study is a multicenter, randomized, parallel-group clinical trial with assessment at the start of the study and four assessment points at 2 weeks, 4 weeks, 6 months, and 12 months after the start of the study. A total of ninety subjects will be enrolled. The participating institutions are Juntendo University Hospital, Juntendo University Shizuoka Hospital, and Juntendo Tokyo Koto Geriatric Medical Center. Patients are required to have no symptoms or mild symptoms that do not bother them at the time of enrollment in the study. Patients who provide informed consent and meet eligibility criteria will be enrolled and will be randomly allocated to the group in which PPI administration is abruptly discontinued (Group A) or the group in which PPI administration is gradually tapered and discontinued (Group B). A schematic of the trial design is presented in Fig. 1.

The study protocol was reviewed and approved by the Juntendo Hospital Certified Review Board (certification no. CRB3180012). This study was registered by the Japan Registry of Clinical Trials (jRCT1031180383) on March 20, 2019. The latest version of the protocol is Version 3.0, which was registered on January 30, 2021. The reason for amendment from Version 1.0 to Version 2.0 on November 3, 2019 was extension of the research period. The reason for amendment from the second version to the third version (Version 3.0) was the change of the data management manager.

### Sample size and power calculation

Power calculations were conducted to determine the required sample size for the primary outcome using Pearson chi-square test for ratio difference. The minimum sample size for each group was identified as 42 to detect a moderate effect size of 0.3 with a power of 0.8 and an alpha of 0.05, based on a previous study [19]. Therefore, the randomized controlled trial will enroll 90 participants (45 in each group) and allow for up to 7% loss to follow-up.

### Eligibility of participants

Patients with gastroesophageal reflux disease (GERD) who have symptoms of heartburn and/or regurgitation will be recruited. To participate in this study, patients are required to meet the following inclusion criteria: (a) the patient is 20 years old or older; (b) the patient had symptoms of heartburn and/or regurgitation before starting oral PPI administration (the presence or absence of symptoms was determined by the interview about the symptoms at the time of starting PPI or by the description of symptoms in the chart at the time of starting PPI); (c) the patient has been taking a PPI (esomeprazole 20 mg, lansoprazole 30 mg, or vonoprazan 20 mg) continuously for more than six months before providing consent to participate in this study; (d) the patient has no symptoms of heartburn and/or regurgitation or has mild symptoms that do not bother the patient; (e) the patient is an outpatient; (f) the patient gives written informed consent to participate in this study. Following are the exclusion criteria: (a) the patient is taking low-dose aspirin, a nonsteroidal anti-inflammatory drug, warfarin potassium and/or a direct oral anticoagulant; (b) the patient is infected by *H. pylori* or has undergone *H. pylori* eradication therapy within the last 1 year; (c) the patient has a history of severe reflux esophagitis such as Los Angeles classification C or D or is currently suffering from severe reflux esophagitis; (d) the patient has an endoscopically confirmed gastric ulcer or duodenal ulcer (both excluding the scarring stage) and is under treatment; (e) the patient currently has a malignant tumor(s); (f) the patient has undergone gastric/esophageal laparotomy or laparoscopic resection; (g) the patient underwent endoscopic treatment of the esophagus/stomach within the past 1 year; (h) the patient has uncontrolled complications.

### Withdrawal of participants

Participants will have the right to withdraw from this study at any time for any reason.

### Intervention

Group A is the group in which PPI administration will be abruptly discontinued. Participants allocated to Group A will stop administration of PPI at the start of the study. Group B is the group in which PPI administration will be gradually tapered and discontinued. Participants allocated to Group B will take half of their regular dose of oral PPI for 2 weeks at the start of the study and then PPI use will be discontinued.

The same kind and same amount of medication which had been prescribed continuously before study enrollment, will be the rescue treatment. If participants experience heartburn and/or regurgitation that bothers them, they can take the rescue treatment without hesitation. Each participant is asked to record daily in a structured diary how much PPI, if any, the participant took each day.

### Randomization

An allocation system will be constructed on the electronic data capture using a Research Electronic Data Capture (REDCap) system [20] and allocation will be performed. Subjects whose eligibility is confirmed, will be randomly assigned to Group A or Group B at a ratio of 1:1 based on the following stratified allocation factor. The stratification factor is gender (male/female).

### Background information and observation items

#### Background information

The background characteristics of participants that will be collected include date of birth, gender, height, weight, medical history, complications, smoking history, and drinking history. The date and the results of the most recent upper gastrointestinal endoscopy including the degree of reflux esophagitis, presence or absence of esophageal hiatal hernia, and gastric mucosal atrophy, will be obtained from the patient’s chart. *H. pylori* infection status (uninvestigated, uninfected, after eradication), serum gastrin level, kinds of PPI and the duration of oral PPI administration, will be obtained from the patient’s chart. Information on concomitant medications will be obtained from the patient’s chart. Participants will be asked to fill out the Hospital Anxiety and Depression Scale questionnaire [21].

#### Rescue treatment

Patients are instructed that if the patient develops heartburn or regurgitation that bothers him/her, the patient can take the rescue treatment, which was the dose of PPI that the patient was taking before the start of this study. Each patient is given a structured diary and is asked to record daily how much PPI, if any, the patient took each day. At the 4-week, 6-month and 12-month visits, the doctor will review the patient’s diary and confirm with the patient how many days the patient took the rescue treatment during the 28-day period prior to that visit.

#### Symptom questionnaire

Participants will be asked to fill out the Symptom questionnaire of the Global Overall Symptom (GOS) scale and the Frequency Scale for the Symptoms of GERD (FSSG) at the start of the study and at 2 weeks, 4 weeks, 6 months, and 12 months after the start of the study. The GOS scale is a 7-point scale as follows [22]: 1, No problems; 2, Minimal problems; 3, Mild problems; 4, Moderate problems; 5, Moderately severe problems; 6. Severe problems; 7, Very severe problems. Participants are asked to grade the overall severity of the following eight symptoms: stomach pain, heartburn, regurgitation, postprandial fullness, nausea, belching, early satiety, and bloating. The FSSG consists of 12 items [23]: seven items are related to reflux symptoms, while five items are related to dyspeptic symptoms. The scores are calculated according to the frequency of the symptoms as follows: 0, never; 1, occasionally; 2, sometimes; 3, often; and 4, always.

### Statistical analysis

The population analyzed in this study will be the population analyzed for intention-to-treat in all randomized cases. In addition, in order to confirm the robustness of the test results, the target population that conforms to the test protocol, that is, the per-protocol set, will also be analyzed.

The significance level is set at 5% in a two-tailed test and the confidence interval is 95%. Regarding the background of the study subjects, continuous variables will be expressed as the mean and standard deviation, and categorical variables will be expressed as the number of patients and proportion. For the primary and secondary endpoints, differences between groups will be assessed using Chi-square tests with 95% confidence intervals and p-values. Regarding safety, each item will be expressed as the number of patients and proportion in each group, and Fisher's exact test will be used to compare the proportions between groups.

Factors contributing to failure of PPI discontinuation will be investigated as additional analyses.

If there are data at the 6-month point, which is a primary endpoint, the data will be used even if data of other assessment points are missing. Missing values will not be complemented.

### Outcomes

#### Primary outcome

It will be judged that a PPI is successfully discontinued if the number of days of rescue treatments performed during the 28-day period before a particular visit is less than 8 days. The proportion of patients who successfully discontinued the PPI at 6 months after the start of the study will be compared between Group A and Group B.

#### Secondary outcome

The proportion of patients who successfully discontinued the PPI at 12 months after the start of the study will be compared between Group A and Group B.

#### Safety evaluation

The presence of findings suggestive of gastrointestinal bleeding such as melena, hematochezia, and hematemesis, and gastrointestinal perforation such as peritoneal irritation as adverse events will be evaluated by interview at the visit or by telephone interview.

The proportion of patients with adverse events will be compared between Group A and Group B.

### Quality control and quality assurance

#### Monitoring and auditing

This study will be monitored and audited by a person who belongs to the Juntendo Clinical Research and Trial Center, who is independent from the sponsor and competing interests. Monitoring will be done annually. Audits will be conducted as necessary and will be conducted at the end of the study.

#### Data management

The data management of this study will be carried out by the data management manager appointed by the principal investigator using REDCap.

### How consent will be obtained from patients

The principal investigator or a co-investigator will fully explain the content of the study based on the explanatory/consent document to patients who meet the eligibility criteria. The patient is given sufficient time to think about whether or not to participate in this study. The principal investigator or co-investigator will obtain written informed consent from patients who agree to participate in the study.

### Confidentiality

Each enrolled participant will be assigned a research identification number that is not related to his/her personal information, and sufficient consideration will be given to protect the confidentiality of the participants.

### Compensation for health damage

This study will be covered by clinical research insurance in preparation for compensation liability in the event of health damage to the participants.

### Publication of results

The results obtained in this study will be presented at the Meeting of the Japanese Society of Gastroenterology and will be published as an article in an overseas journal in the field of gastroenterology. In either case, the personal information of the participants will not be published.

## Discussion

Establishing a strategy to safely and securely discontinue PPI is considered very meaningful. Another meaningful point of this study is that by explaining this study, patients will understand that a PPI is a drug that must be taken while considering its necessity.

We predict that the proportion of patients who successfully discontinue PPI will be higher in the group in which PPI administration is gradually tapered and discontinued than in the group in which PPI administration is abruptly discontinued. On the other hand, we expect that many participants may succeed in discontinuing PPI regardless of the discontinuation strategy due to the explanation that discontinuation is necessary.

By comparing the background characteristics between participants who are able to discontinue PPI and those who are not able to discontinue PPI, it may be possible to clarify factors related to the difficulty of PPI discontinuation.

## Trial status

The study’s start date was March 12, 2019. At the time of this manuscript submission, recruitment for this study is ongoing. The proposed end date is August 31, 2023 (the end of follow-up).

## Data Availability

The datasets used and/or analyzed during the current study will be available from the corresponding author on reasonable request.

## Abbreviations

PPI: Proton pump inhibitor
PCAB: Potassium ion-competitive acid blocker
*H. pylori*: *Helicobacter pylori*
NERD: Non-erosive gastroesophageal reflux disease
FD: Functional dyspepsia
GERD: Gastroesophageal reflux disease
REDCap: Research Electronic Data Capture
GOS: Global Overall Symptom
FSSG: Frequency Scale for the Symptoms of GERD
